# Developing an Integrated Municipal Environmental Health Framework for Communicable Disease Surveillance and Prevention in South Africa: A Mixed-Methods Study Protocol

**DOI:** 10.3390/tropicalmed11020056

**Published:** 2026-02-17

**Authors:** Ledile Francina Malebana, Maasago Mercy Sepadi, Matlou Ingrid Mokgobu

**Affiliations:** 1 Environmental Health Department, Faculty of Science, Tshwane University of Technology, Pretoria 0183, South Africa; sepadimm@tut.ac.za; 2 Office of the Campus Rector, Tshwane University of Technology, Pretoria 0183, South Africa; mokgobumi@tut.ac.za

**Keywords:** environmental health, communicable disease surveillance, disease prevention, framework development, municipal health services, South Africa

## Abstract

Communicable diseases remain a significant public health burden in South Africa, particularly where environmental determinants of health intersect with fragmented surveillance systems. Environmental Health Practitioners (EHPs) are legally mandated to implement the surveillance and prevention of communicable disease services at the municipal level. However, this function is inconsistently operationalised and often remains reactive (outbreak-driven), with limited integration into broader national surveillance systems. This study protocol outlines a mixed-methods investigation to develop a practical framework to strengthen the communicable disease surveillance and prevention function within Environmental Health Services in South Africa. The study will assess existing guiding tools, operational practices, and intersectoral collaboration mechanisms supporting surveillance across metropolitan and district municipalities. Quantitative data will be collected through a national survey of EHPs, while qualitative data will be generated through key informant interviews with national stakeholders, focus group discussions with municipal health managers, and a targeted review of municipal documents. Quantitative data will be analysed using descriptive and inferential statistics, while qualitative data will be thematically analysed and triangulated across data sources. The expected outcome is an integrated framework that clarifies roles, strengthens data flow, and promotes proactive, coordinated surveillance and prevention of communicable diseases within environmental health. The developed framework is anticipated to inform policy discussions and may contribute to efforts aligned with Sustainable Development Goal 3, Target 3.3, on reducing communicable disease burdens, by strengthening municipal communicable disease surveillance and prevention.

## 1. Introduction

Communicable diseases continue to represent a significant public health challenge globally and to impose a disproportionate burden on low- and middle-income countries, where environmental conditions, socio-economic inequalities, and health system constraints intersect [1,2]. Effective communicable disease surveillance and prevention are foundational to public health systems, protecting population health, reducing morbidity and mortality, and supporting Sustainable Development Goal 3 target 3.3, which requires countries to reduce infectious disease epidemics by 2030. Robust surveillance systems support early outbreak detection, monitoring of disease trends, and evidence-based public health decision-making, while mitigating the broader social and economic impacts of disease outbreaks, as highlighted during the COVID-19 pandemic [3]. These functions rely on the systematic use of health-related data to inform timely interventions [4]. Within this context, Environmental Health Practitioners (EHPs) contribute by generating and utilising surveillance data related to environmentally induced diseases and their determinants to support public health protection.

Given this context, environmental determinants such as water quality, sanitation, food safety, waste management, housing conditions, and vector control play a critical role in the transmission of communicable diseases, for example, cholera outbreaks caused by the presence of *Vibrio cholerae* in drinking water, or malaria disease caused by Anopheles mosquito bites [5]. Therefore, Environmental Health Services form an integral component of a comprehensive disease surveillance and prevention systems. In recognition of this, Environmental Health Practitioners (EHPs) are mandated, both internationally and nationally, to contribute to communicable disease surveillance and prevention as part of environmental health practice [2]. In South Africa, this mandate is explicitly articulated in Section 5 of the regulations defining the scope of practice of EHPs under the Health Professions Act 56 of 1974, which assigns EHPs responsibilities related to disease prevention, control, and the collection and dissemination of epidemiological information [6].

At a governance level, communicable disease surveillance and prevention are embedded within the Municipal Health Services (MHS) function, as outlined in the National Health Act 61 of 2003 and the Municipal Structures Act 117 of 1998 [7]. Moreover, the districts and metropolitan municipalities were assigned under sections 156, schedule 4, and 5 of Part B of the Constitution of the Republic of South Africa, 1996, to render the MHSs as a component of the environmental health profession [8]. This positioning places Environmental Health Services (EHSs) at the interface between communities, municipal governance structures, and the broader public health system.

Despite this legislative mandate, evidence suggests that communicable disease surveillance and prevention within MHSs remain inconsistently implemented across South African municipalities and are predominantly reactive (outbreak-driven), meaning that surveillance activities are initiated primarily in response to outbreaks rather than embedded within routine, proactive monitoring systems. Such practices limit opportunities for early detection and timely prevention [9]. Furthermore, the structural features of the health system, including fragmented reporting lines and weak coordination between municipal Environmental Health Services and primary health care (PHC) structures, further constrain effective surveillance. Additionally, the absence of clear operational guidance for routine surveillance activities undermines the integration of environmental health contributions into a broader public health decision-making [10].

Public health emergencies like the 2017–2018 listeriosis outbreak have exposed systemic weaknesses in communicable disease surveillance [11]. While these events temporarily improved intersectoral collaboration and data sharing, these mechanisms were not institutionalised through standardised frameworks or policies that guide environmental health practice [12,13]. As a result, the improvements made during crises have not become part of sustained, routine surveillance systems at the municipal level.

Although disease surveillance is recognised as a core public health function and a critical component of PHC, limited empirical research has examined how EHPs operationalise communicable disease surveillance in practice, the types of data they collect, how information flows across governance levels, and the institutional and operational barriers that impede proactive surveillance [4,10]. Addressing these gaps requires a systematic examination of current practices and the development of a coherent framework to guide the implementation of communicable disease surveillance and prevention within EHSs. With that said, this study aims to develop an integrated framework to strengthen communicable disease surveillance and prevention within municipal Environmental Health Services in South Africa, thereby examining existing surveillance guidance tools, surveillance activities, practices, and the challenges that influence implementation across metropolitan and district municipalities.

Therefore, a mixed-methods investigation is critical for informing the development of a practical framework to support proactive municipal-level communicable disease surveillance and prevention.

### 1.1. Conceptual Framework of the Study

This study is informed by a systems-based approach to communicable disease surveillance, drawing on the World Health Organization health systems framework and integrated disease surveillance principles, which conceptualise surveillance as a continuous, coordinated public health function embedded within routine primary health care (PHC) service delivery rather than a discrete, outbreak-driven activity [4]. Within this approach, surveillance systems depend on the effective interaction of institutional structures, operational processes, and information flows across multiple levels of the health system, with PHC serving as a foundational platform for prevention, early detection, and response.

Environmental Health Services are conceptualised as a core municipal component of community-oriented PHC and a critical subsystem within South Africa’s public health surveillance architecture. Environmental Health Practitioners, operating at the municipal level, are strategically positioned to contribute to the early detection and prevention of communicable diseases through routine environmental monitoring of disease determinants and regulatory functions [2,11]. This role, as it is underpinned by national legislation, includes the collection, analysis, and dissemination of surveillance data to support PHC-based disease prevention and health promotion [6,7].

However, empirical evidence and national experience indicate that the effectiveness of this role is constrained by fragmented governance arrangements, limited operational guidance, and weak functional integration between EHSs, PHC services, and national disease surveillance systems [10]. These challenges became particularly evident following the devolution of EHSs to district and metropolitan municipalities, which disrupted reporting lines and contributed to inconsistent implementation of surveillance functions across municipalities.

The proposed framework is structured around three interrelated components, consistent with the public health systems and surveillance literature, and is explicitly anchored within PHC. The first component comprises structural inputs, including legislative mandates, institutional arrangements, human resource capacity, surveillance infrastructure, and guiding tools such as policies, norms, standards, and standard operating procedures [1,9]. In the South African context, these inputs include the regulatory framework governing environmental health practice, MHS mandates, PHC policies, and national communicable disease surveillance policies coordinated through the National Institute for Communicable Diseases (NICD). Accordingly, the first study objective examines the availability and alignment of these inputs within municipal Environmental Health Services.

The second component focuses on operational processes within PHC-oriented surveillance, including routine environmental health inspections, case investigation, data collection, analysis, reporting, feedback mechanisms, and intersectoral collaboration in line with the second and third objectives of the study [4,11]. NICD surveillance guidelines and notifiable disease reporting systems emphasise the importance of timely data flow, coordination between local and national levels, and integration of environmental and clinical surveillance data to support early detection and response [12]. Experiences during the COVID-19 pandemic further highlighted the critical role of Environmental Health Practitioners as actors aligned to PHC through contact tracing support, environmental investigations, risk communication, and enforcement of public health measures, demonstrating the potential for strengthened collaboration when clear guidance and coordination mechanisms are in place [12,13].

The third component addresses intended outcomes related to strengthened detection, prevention, and public health decision-making, corresponding to the fourth objective, and aligned with PHC objectives, including improved early detection of communicable disease risks, strengthened preventive action, coordinated outbreak response, and enhanced accountability across governance levels. Evidence from the COVID-19 response indicated that when surveillance functions are clearly defined, supported by operational guidance, and integrated across relevant sectors, EHSs and PHC systems can meaningfully contribute to national surveillance objectives and public health resilience [14]. Conversely, the absence of sustained linkages with PHC frameworks following emergency periods results in a reversion to reactive, case-driven practices.

The framework development in this study will be informed by empirical evidence generated through a mixed-methods approach that incorporates the perspectives of EHPs, municipal managers, national stakeholders, and surveillance documents. By mapping current practices against legislative requirements, PHC principles, NICD surveillance guidance, and public health surveillance frameworks, the study identifies gaps in coordination, data flow, and operational guidance that limit the capacity for proactive communicable disease surveillance and prevention [10,12].

By explicitly positioning Environmental Health Practitioners as active contributors to community-oriented, prevention-focused primary health care rather than peripheral responders, the proposed framework aims to strengthen functional integration between municipal EHSs, PHC services, and national surveillance systems. This approach supports a shift from reactive outbreak management towards anticipatory and preventive environmental health practice, aligned with PHC principles and Sustainable Development Goal 3.3, which calls for ending epidemics of major communicable diseases by 2030 [15].

This framework represents a conceptual and operational synthesis informed by empirical findings from the study and is not intended as a validated implementation model, as its applicability and effectiveness will require future evaluation through implementation and monitoring within the municipal settings.

### 1.2. Problem Statement

Communicable disease surveillance and prevention within municipal settings relies on the effective use of routinely generated data to inform timely preventive and control measures [10]. However, the mandated functions are frequently implemented in response to outbreaks, rather than as part of day-to-day practice. Therefore, this gap highlights persistent challenges in translating legislative intent into proactive, systemic surveillance at the municipal level [11].

Furthermore, there remains limited empirical clarity on how communicable disease surveillance and prevention are routinely operationalised within municipal health services. In particular, gaps persist in understanding how governance arrangements, reporting pathways, and coordination mechanisms between Environmental Health Services, primary health care, and national surveillance structures, such as those coordinated by the National Institute for Communicable Diseases, shape the integration of environmental health disease surveillance into routine public health practice [12].

Furthermore, there is limited clarity regarding the types of surveillance data collected by EHPs, the methods used for data collection and reporting, and the mechanisms through which environmental health information is integrated into broader communicable disease surveillance systems. Although national surveillance frameworks and notifiable disease reporting systems exist, they do not provide sufficient operational guidance specific to EHSs at the municipal level [10,13].

The COVID-19 pandemic temporarily strengthened collaboration between EHSs and PHC through emergency guidance and outbreak response protocols; however, these mechanisms were largely crisis-driven and not sustained through permanent frameworks or standard operating procedures [12,14]. As a result, routine communicable disease surveillance within EHSs remains inconsistent across municipalities and predominantly focused on control rather than prevention.

The absence of a coherent framework to guide the implementation of communicable disease surveillance and prevention within EHSs has contributed to variability in practice, limited data integration, and missed opportunities for early detection and preventive action. Addressing this gap requires a systematic assessment of existing practices and the development of an integrated framework that supports proactive, coordinated, and sustainable surveillance and prevention at the municipal level.

### 1.3. Aim of the Study

This study aims to develop an operational framework to strengthen the implementation of communicable disease surveillance and prevention within EHSs in South Africa.

### 1.4. Objectives of the Study

The specific objectives of the study are to

(1) Identify existing guiding tools, including legislation, policies, norms, standards, and standard operating procedures, that inform communicable disease surveillance and prevention within EHSs across metropolitan and district municipalities.
(2) Determine the operational activities carried out by EHPs in relation to communicable disease surveillance and prevention as stipulated in the regulations defining the scope of practice.
(3) Assess the types of surveillance data collected by EHPs, the methods used for data collection, and the sources from which such data are obtained for communicable disease prevention.
(4) Identify structural, operational, and coordination-related challenges that hinder the effective implementation of communicable disease surveillance and prevention within EHSs.
(5) Develop an integrated framework to guide the proactive implementation of communicable disease surveillance and prevention within EHSs in South Africa.

### 1.5. Research Questions

The study seeks to address the following research questions:

(1) What guiding tools, policies, and regulatory instruments inform communicable disease surveillance and prevention within municipal EHSs?
(2) How are communicable disease surveillance and prevention activities operationalised by EHPs in metropolitan and district municipalities?
(3) What surveillance data are generated through EHSs, and how do data collection methods and sources support communicable disease surveillance?
(4) What institutional, operational, and systemic challenges hinder effective implementation of communicable disease surveillance and prevention within EHSs?
(5) How can an integrated framework be developed to strengthen proactive communicable disease surveillance and prevention within municipal EHSs in South Africa?

## 2. Materials and Methods

### 2.1. Study Design

This study will employ a cross-sectional, convergent mixed-methods design to examine the implementation of communicable disease surveillance and prevention within EHS across South African municipalities at a defined point in time. A mixed-methods approach is appropriate for this study, as it allows for integration of quantitative data on current practices with qualitative insights into contextual, operational, and coordination-related challenges [16].

The quantitative component will provide breadth by assessing surveillance activities, including the data collection, analysis, and dissemination practices, and implementation patterns among EHPs across metropolitan and district municipalities. The qualitative component will provide depth through interviews, focus group discussions, and document reviews, enabling an in-depth exploration of guiding tools, intersectoral collaboration, and systemic constraints influencing communicable disease surveillance and prevention [17].

This design aligns with the study objectives and supports triangulation of findings, thereby strengthening the credibility and applicability of the proposed framework.

### 2.2. Study Setting

The study will be conducted in district and metropolitan municipalities across the nine provinces of South Africa, where EHPs are responsible for delivering MHSs. South Africa is situated at the southern tip of the African continent, sharing borders with six other countries, such as Botswana, Namibia, Zimbabwe, Mozambique, and Eswatini, while the Kingdom of Lesotho is entirely landlocked within its territory. The country has a total surface area of approximately 1.22 million square kilometres, making it the 25th largest country in the world.

**Figure 1:**
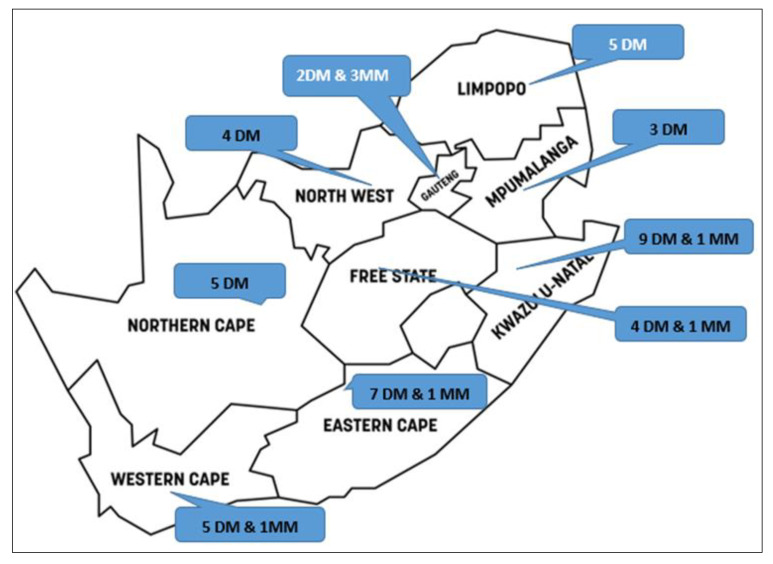
The distribution and number of district municipalities (DMs) and metropolitan municipalities (MMs) per province in South Africa.

To contextualise the governance structures within which EHS are implemented at municipalities, this study considers the municipal configuration of South Africa. Therefore, the country comprises eight metropolitan municipalities and forty-four district municipalities, which together constitute the primary governance structures responsible for delivering EHSs [8]. With that said, Figure 1 illustrates the distribution of these municipal structures across all provinces.

Furthermore, to support in-depth qualitative research, six municipalities will be purposively selected for document review and stakeholder engagement. This selection will be based on specified criteria, including municipal category, geographic location across states, and variation in service delivery, as further detailed in the sampling strategy section. The participants will be further selected based on their professional roles, level of engagement in surveillance activities, and institutional knowledge relevant to the study objectives. These will include

- Two metropolitan municipalities (representing predominantly urban and mixed urban–rural contexts);
- Four district municipalities representing rural, semi-rural, and semi-urban settings.

This selection enables the exploration of surveillance practices across diverse municipal contexts while remaining feasible within the study’s logistical constraints.

### 2.3. Study Population

The study population will comprise EHPs registered with the Health Professions Council of South Africa (HPCSA) as independent practitioners and currently rendering MHS in district and metropolitan municipalities.

In addition, key informants will include officials from national and sectoral institutions responsible for policy development, regulation, and coordination of communicable disease surveillance and EHSs. These include representatives from

- The National Department of Health (environmental health, communicable diseases, and PHC directorates);
- The South African Local Government Association (SALGA), Municipal Health Services Component.

Municipal environmental health managers will also participate in the qualitative components of the study, given their oversight roles in operational planning, coordination, and reporting.

Lastly, the population for the document review comprises 52 metropolitan and district municipalities responsible for implementing municipal health services, including communicable disease surveillance and prevention. These municipalities constitute the units of analysis for reviewing relevant policies, operational documents, including SOPs, outbreak response protocols, surveillance guidelines, and municipal by-laws.

### 2.4. Sampling Strategy and Sample Size

**Figure 2:**
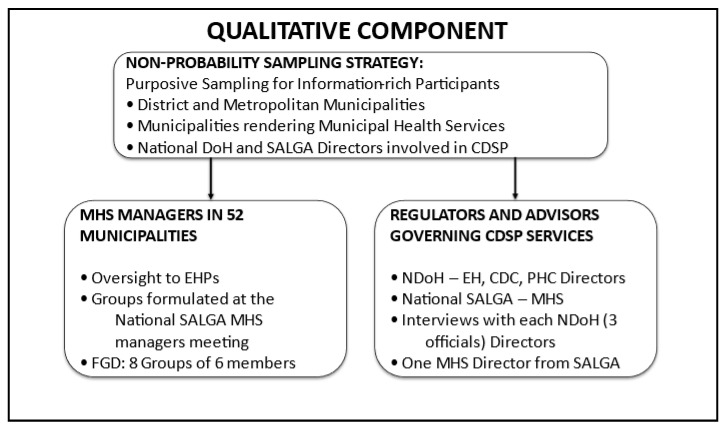
Qualitative sampling strategy and sample size.

In general, the study applies a combination of probability and non-probability sampling, where the quantitative approach used a stratified sampling technique to obtain the participation of EHPs across the country, while the qualitative approach used the purposive sampling strategy to obtain participants for the interviews, focus groups, and document reviews [16]. Subsequently, the use of both strategies reflects the study’s mixed-methods design, whereby probability sampling ensured representativeness for quantitative analysis, while purposive non-probability sampling enabled in-depth qualitative insights from information-rich cases. This is illustrated in Figure 2, which shows the proposed sampling strategy.

#### 2.4.1. Quantitative Component: Environmental Health Practitioners

The quantitative component will target Environmental Health Practitioners across all nine provinces of South Africa. The total population of registered EHPs rendering Municipal Health Services is estimated at approximately 4226.

The minimum sample size was calculated using Yamane’s finite-population sampling formula, assuming a 95% confidence level and 5% margin of error [17].

The formula is as follows:

$$n=\frac{N}{{1+N(e}^{2})}$$

where *n* is the required sample size, *N* is the population size (4226), and *e* is the margin of error (0.05). Based on these parameters, a minimum of 365 Environmental Health Practitioners will be required.

To ensure adequate representation across provinces, a proportional stratified random sampling approach was applied, where each province constituted a stratum, and the number of EHPs selected per province was determined using proportional allocation:

*nᵢ* = *n* × (*Nᵢ*/*N*)

where *n_i_* is the required sample size for province *i*, *n* is the total sample size (365), *N_i_* is the number of eligible EHPs in province *i*, and *N* is the total EHP population. Simple random sampling without replacement was then used to select participants within each stratum, as presented in Table 1. The table illustrates the proportional allocation of the Environmental Health Practitioner (EHP) sample across South African provinces based on population size and municipal distribution. Specifically, the total population of EHPs was stratified by province and by district or metropolitan municipality. The total sample size (*n_i_*) was then allocated proportionally to each stratum based on its share of the national EHP population using proportional allocation. This approach ensured that strata with larger EHP populations contributed a proportionally greater number of respondents, while smaller strata were adequately represented.

#### 2.4.2. Qualitative Component

For the qualitative component, purposive sampling will be used to recruit participants directly involved in communicable disease surveillance and prevention.

This will include

- Key informant interviews with each official from the relevant NDoH Directorates, such as EH, CDC, PHC directors, and the national SALGA MHS director (four participants);
- Focus group discussions with environmental health managers from all districts and metropolitan municipalities, which are eight groups of six members.

Participants will be selected based on their role, experience, and involvement in surveillance and prevention activities. The National SALGA MHS managers’ platform will be used to establish and convene the focus group discussions during their meeting. Data collection will continue until thematic saturation is achieved. Consistent with this, Figure 2 illustrates the sampling strategy and sample sizes for the interviews and focus group discussions.

#### 2.4.3. Document Review of Municipal Policies, Standard Operating Procedures, and Surveillance-Related Records from the Selected Municipalities

For the document review component, the researcher will employ a stratified purposive sampling strategy combined with a proportionate fixed fraction (10%) sampling approach [17]. This strategy was selected to ensure that the sampled municipalities were both representative of diverse service delivery contexts and information-rich, thereby supporting an in-depth analysis of the implementation of communicable disease surveillance and prevention services in environmental health.

The sample size was calculated as follows:

*n* = *N* × 0.10

where

- *n* = sample size;
- *N* = total population size.

*n* = 52 × 0.10 = 5.2 ≈ 6 *municipalities*

**Figure 3:**
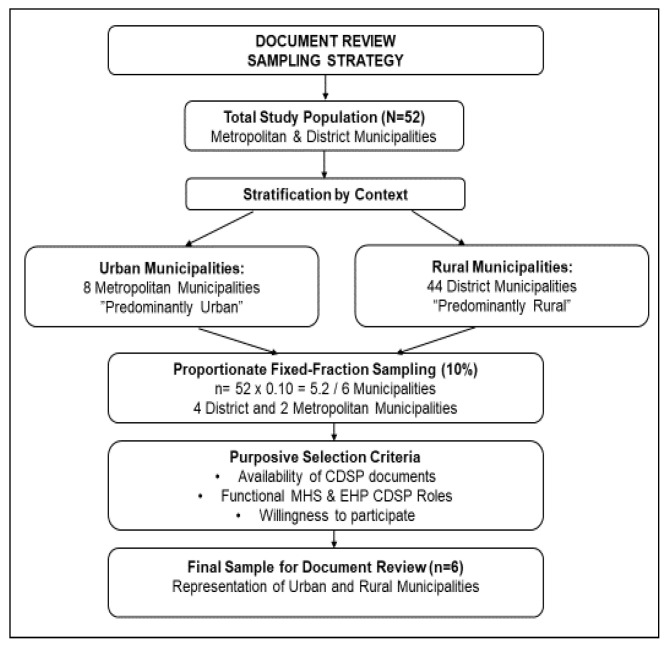
Document review sampling strategy and sample size.

Accordingly, six municipalities will be considered, as illustrated in Figure 3, for the document review component of the study, including adequate representation across both strata, to strengthen the comparative analysis between rural and urban settings.

#### 2.4.4. Inclusion and Exclusion Criteria

The inclusion criteria are as follows: Districts or metropolitan municipalities in South Africa that currently render MHSs. EHPs registered with the Health Professions Council of South Africa (HPCSA) and employed within MHSs in district or metropolitan municipalities. The directors or managers responsible for EH, CDC, and PHC within NDoH, and the national MHS portfolio at SALGA, and municipal managers overseeing EHPs. Lastly, relevant policies, guidelines, norms and standards, standard operating procedures, and municipal records directly related to communicable disease surveillance and prevention.

The exclusion criteria are as follows: Municipalities that do not render MHSs. Practitioners who are not registered with HPCSA or who are not employed within MHSs. Managers or officials whose roles do not relate to communicable disease surveillance, EHSs, or MHSs. Lastly, documents unrelated to this field of study or outside the scope of EHSs.

**Figure 4:**
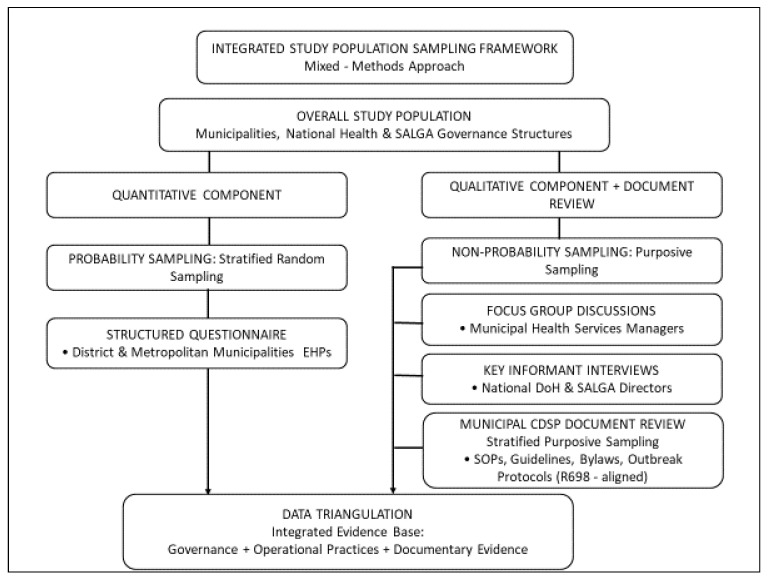
Integrated population sampling strategies.

In summary, the qualitative sampling framework illustrates purposive selection of municipal and national participants, integrated with stratified purposive document-review sampling for the study. Therefore, Figure 4 illustrates the integration of the overall sampling strategies used in the study’s mixed-methods approach.

### 2.5. Data Collection Methods

**Figure 5:**
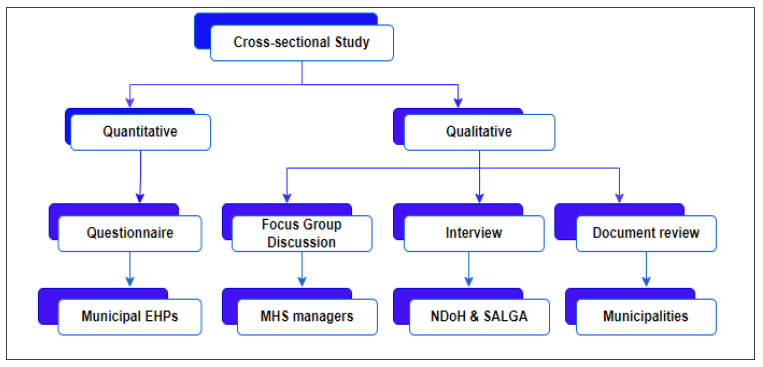
Flow diagram of the research methodology with the study population.

Four complementary data collection methods will be employed to ensure depth and breadth of information and to support triangulation (Figure 5).

#### 2.5.1. Structured Questionnaire Survey

A structured, self-administered online questionnaire (File S1) will be distributed to Environmental Health Practitioners online through a secure Google Forms (Google LLC, Mountainview, CA, USA) link shared via the National SALGA MHS forum. The platform was selected to facilitate national reach and accessibility, with data collection configured to exclude personally identifiable information and access restricted to the research team in accordance with ethical approval and data protection requirements. Therefore, the tool will collect quantitative data on

- Communicable disease surveillance and prevention activities;
- Types and sources of surveillance data collected;
- Methods of data collection, analysis, and reporting;
- Perceived challenges affecting implementation.

The questionnaire was informed by legislative requirements, NICD surveillance guidance, and the relevant literature on public health surveillance [10,12]. The data gathered through the questionnaire will assist the researcher in identifying and delineating operational activities in CDSP by municipalities (objectives 2 and 3). Furthermore, the questionnaire was piloted to strengthen its reliability, validity, and feasibility, and amended accordingly to ensure that participants understood the questions.

#### 2.5.2. Key Informant Interviews

Semi-structured interviews will be conducted with key informants from national and sectoral institutions involved in disease surveillance to explore

- Guiding policies and surveillance frameworks;
- Coordination mechanisms between EHSs and PHC;
- Data integration and reporting systems;
- Gaps in current surveillance structures.

The semi-structured interview guide was developed in line with study objective 1, drawing on the relevant literature, regulatory frameworks, and the roles of EHPs in communicable disease surveillance and prevention. Subsequently, the guide was piloted with relevant officials not included in the main study to assess clarity, relevance, and sequencing of questions, and was amended accordingly. This interview guide will be used to maintain consistency while allowing flexibility for in-depth exploration of governance issues (Objective 1) within the EH CDSP.

#### 2.5.3. Focus Group Discussions

Focus group discussions will be conducted with environmental health managers from district and metropolitan municipalities to examine

- Operational experiences in implementing communicable disease surveillance;
- Coordination challenges within and across sectors;
- Practical constraints affecting proactive surveillance and prevention.

These discussions will enable comparison of experiences across municipalities and support identification of common systemic challenges (objective 4).

#### 2.5.4. Document Review

A structured document review will be conducted to analyse

- Municipal standard operating procedures;
- Surveillance guidelines, and reports;
- Reporting tools;
- Relevant policy and legislative documents.

The document review extraction matrix was developed for systematic review of the listed documents, to assess the presence, scope, and alignment of governance arrangements, guiding tools, and operational activities. This supports objective 1 of assessing existing guiding tools and aligning them with legislative and national surveillance requirements.

### 2.6. Ethical Considerations

Ethical approval for this study was obtained from the Tshwane University of Technology Human Research Ethics Committee. The study will be conducted in accordance with internationally accepted ethical principles for research involving human participants, including respect for autonomy, beneficence, non-maleficence, and justice.

Permission to conduct the study was sought from the South African Local Government Association (SALGA) on behalf of participating district and metropolitan municipalities, as well as from the National Department of Health (NDoH) for participation of officials from relevant directorates. All participants will receive an information leaflet outlining the purpose, procedures, potential risks, and benefits of the study.

Participation will be voluntary, and written informed consent will be obtained before data collection [17]. Participants will be informed of their right to decline participation or withdraw from the study at any stage without penalty. No personal identifiers will be collected, and confidentiality will be maintained by using coded identifiers. Data will be securely stored and accessed only by the research team for academic purposes.

### 2.7. Data Analysis

Quantitative data will be analysed using IBM SPSS Statistics version 29 (IBM Corporation, Armonk, NY, USA). Descriptive statistics will be used to summarise surveillance activities, data collection practices, and implementation patterns. Inferential analyses, including chi-square tests to examine associations between categorical variables, correlation analysis to assess relationships between key surveillance variables, and logistic regression analysis to explore predictors of engagement in surveillance activities. Moreover, the cross-tabulations and association tests will be conducted where appropriate to explore differences across municipal contexts. The analysis of the questionnaire data addresses objectives 2 and 3 of the study, along with their respective study questions.

Qualitative data from interviews, focus group discussions, and document reviews will be analysed thematically using ATLAS.ti version 2024 (Lumivero, LLC, Berlin, Germany). An inductive coding approach will be applied to identify patterns and categories emerging from the data, followed by organisation of codes into themes aligned with the study objectives and the inputs-process-outcomes framework components. Furthermore, the interview data will primarily address objective 1 by exploring the governance arrangements and guiding principles, supported by evidence from the municipal document review. On the other hand, the focus group discussions will be analysed to address objective 4 with emphasis on identifying operational challenges and contextual constraints affecting implementation across municipalities.

### 2.8. Data Integration and Triangulation

Findings from the quantitative and qualitative components will be analysed separately and integrated during the interpretation phase through triangulation. Data from questionnaires, interviews, focus groups, and document reviews will be compared to identify convergences, divergences, and complementary insights [18].

These integrated findings will subsequently be synthesised and mapped onto the input-process-outcomes framework to inform the development of the proposed framework, thereby ensuring that it is grounded in empirical evidence, reflects diverse stakeholder perspectives, and addresses both structural and operational dimensions of communicable disease surveillance and prevention.

In addition, these integrations and triangulations serve as quality-assurance measures that enhance the study’s credibility, methodological rigour, and trustworthiness.

## 3. Anticipated Study Outcomes

The results will be presented according to the study objectives and questions provided.

The literature emphasised that surveillance of communicable diseases is the first and most critical step in addressing public health challenges and preventing communicable diseases. Given that, communicable disease surveillance and prevention in South Africa is the mantra of Environmental Health Services [19]. It is one of the core functions of environmental health, as provided for in the Health Professions Act, 56 of 1974 [5]. Based on the legislative mandate outlined in Section 5 of the regulations defining the scope of practice of Environmental Health Practitioners. The study is expected to identify gaps between prescribed communicable disease surveillance and prevention functions and routine practices within municipal health services. Specifically, the findings are anticipated to reveal limited availability of clear, standardised guidelines for implementing communicable disease surveillance functions, despite the existence of broader environmental health norms and standards. The study is further expected to demonstrate variability in how surveillance and prevention activities are operationalized across municipalities, highlighting the need for a more explicit and standardised framework to support consistent implementation of these functions [18]. Consequently, all functions of Environmental Health Practitioners as stipulated in the Health Professional Act are covered within those standards, except the disease surveillance function [18]. The results of the study are expected to indicate the need to further investigate and review standard operating procedures for CDSP services across the country, by strengthening communication and collaboration with other relevant stakeholders affected by and interested in the surveillance of communicable diseases [20].

Additionally, the quantitative component is expected to highlight gaps in the types of surveillance data collected by EHPs and inconsistencies in reporting mechanisms and feedback processes, while qualitative findings are anticipated to reveal structural and operational barriers, including unclear operational guidance, fragmented reporting lines, limited integration with PHC services, and resource constraints at the municipal level.

By integrating findings across data sources, the study is expected to inform the development of a practical and contextually relevant framework that clarifies roles, strengthens data flow, and promotes proactive communicable disease surveillance and prevention within EHSs. The framework is expected to support improved coordination among municipal, provincial, and national stakeholders and to enhance EHPs’ contributions to national surveillance objectives.

### Study Dissemination

The researchers intend to disseminate relevant research findings to research participants, as well as other individuals, institutions, authorities, and researchers interested in the research outcomes. As appropriate, one or a combination of the following methods will be applied to disseminate the research findings during or at the end of the study: media coverage, press releases, flyers, posters, brochures, research briefs, policy briefs, newsletters, community publications, university website, local industry events, seminars, conferences, and community meetings. In any of the above instances, the University’s approval will be obtained.

## 4. Limitations and Delimitations of the Study

This study has several anticipated limitations that will be considered during the interpretation of the findings. First, the cross-sectional design limits the ability to assess changes in surveillance practices over time or to establish causal relationships. Consequently, quantitative findings will be interpreted descriptively and analytically, focusing on associations and patterns rather than causation. Second, reliance on self-reported data from Environmental Health Practitioners may introduce reporting bias, which will be mitigated through triangulation with qualitative interviews, focus group discussions, and document review to enhance credibility and contextual validity.

Additionally, the qualitative component will involve purposive sampling of municipalities, which may limit the generalisability. Findings from this component will therefore be interpreted analytically, with emphasis on contextual variation and transferable insights that inform framework development rather than representativeness. However, the inclusion of diverse metropolitan and district municipalities is expected to support robust interpretation at a system level and to provide sufficient contextual variation to support the development of the framework.

Finally, the study is deliberately delimited to communicable disease surveillance and prevention within Environmental Health Services in district and metropolitan municipalities. Other municipal health service functions and contexts in which Environmental Health Practitioners operate at the local or provincial level were not examined. The study primarily focuses on formally documented policies, norms, standards, and procedures, while informal practices were explored only when they emerged from qualitative data.

Despite these limitations, the mixed-methods design and triangulation approach enhance the credibility and applicability of the findings.

Lastly, the developed framework is positioned as an evidence-informed model intended to guide practice and policy rather than as a validated intervention or implementation tool. Therefore, it will require future evaluations, including potential pilot testing or comparative assessment across different municipal contexts.

## 5. Conclusions

This study protocol outlines a mixed-methods investigation aimed at strengthening the implementation of communicable disease surveillance and prevention within Environmental Health Services in South Africa through the development of a framework. By systematically examining existing guidance tools, operational practices, data collection mechanisms, and coordination structures, the study seeks to address persistent gaps that limit proactive surveillance at the municipal level.

The proposed framework is expected to contribute to improved integration and implementation of Environmental Health CDSP services within national communicable disease surveillance systems and to support a shift from reactive outbreak management towards anticipatory and preventive practice. In doing so, the study aligns with primary health care principles. It will furthermore contribute to national and global public health priorities, including Sustainable Development Goal 3 target 3.3, which requires countries to end the epidemics of communicable diseases.

Future research will be required to assess the framework’s implementation fidelity, feasibility, and impact on communicable disease surveillance outcomes.

## Figures and Tables

**Table 1:** Sampling of EHP per province/stratum.

Province	No. of District (DM)/Metropolitan Municipalities (MM)	Population Size (*n*)	Estimated No. of EHP Per National Ratio (10%)	Sub-Population (*Nᵢ*)	Sample Size (*nᵢ* = *n* × *Nᵢ*/*N*)
Eastern Cape	Two (2) MM Six (6) DM	6,738,223	673	451	39
Western Cape	One (1) MM Five (5) DM	7,092,527	709	484	42
Northern Cape	Five (5) DM	1,320,924	132	117	10
Kwa-Zulu Natal	One (1) MM Nine (9) DM	11,563,357	1156	839	72
Free State	One (1) MM Four (4) DM	2,913,531	291	371	32
Gauteng	Three (3) MM Two (2) DM	15,882,396	1588	1148	99
North West	Four (4) DM	4,158,730	415	162	14
Mpumalanga	Three (3) DM	4,733,276	473	294	25
Limpopo	Five (5) DM	6,063,742	606	301	26
Total Population (N)	Eight (8) MM Forty-four (44) DM	60,466,705	6043	4226	365

## Data Availability

No new data were created.

## Supplementary Materials

The following supporting information can be downloaded at https://www.mdpi.com/article/10.3390/tropicalmed11020056/s1, File S1: Data collection tools for this mixed-methods study.

## Abbreviations

The following abbreviations are used in this manuscript:

CD	Communicable Disease
CDC	Communicable Disease Control
CDSP	Communicable Disease Surveillance and Prevention
COVID-19	Coronavirus Disease 2019
DM	District Municipality
EHP	Environmental Health Practitioner
EH	Environmental Health
EHS	Environmental Health Services
HPCSA	Health Professions Council of South Africa
FCRE	Faculty of Science Research Ethics Committee
LMICs	Low- and Middle-Income Countries
MHS	Municipal Health Services
MM	Metropolitan Municipality
NICD	National Institute for Communicable Diseases
NDoH	National Department of Health
PHC	Primary Health Care
SA	South Africa
SALGA	South African Local Government Association
SDG	Sustainable Development Goal
SOP	Standard Operating Procedure
SPSS	Statistical Package for the Social Sciences
WHO	World Health Organization
TUT	Tshwane University of Technology
