# Supplementary material for: Developing an Integrated Municipal Environmental Health Framework for Communicable Disease Surveillance and Prevention in South Africa: A Mixed-Methods Study Protocol
Source: Trop Med Infect Dis. 2026 Feb 17;11(2):56. doi: 10.3390/tropicalmed11020056 (PMC12945199; doi:10.3390/tropicalmed11020056)
Supplement: Supplementary file 1 [file tropicalmed-11-00056-s001.zip › tropicalmed-4123145-supplementary.pdf]

STRUCTURED QUESTIONNAIRE WITH ENVIRONMENTAL HEALTH PRACTITIONERS - DISEASES SURVEILLANCE

The objective of this study is to assess disease surveillance and prevention function of EHP, including the types of disease surveillance data, the method used to collect data and the areas where EHPs obtain data for prevention of communicable diseases.

Please complete all the questions and choose the relevant options where required. Where the question does not apply to you, please indicate so by typing not applicable.

* Required

1. Do you agree to participate in this study? *

- ◯ Yes
- ◯ No

2. What is your gender? *

- ◯ Female
- ◯ Male
- ◯ Other

3. In which age (years) category do you fall? *

- ◯ 18 - 20
- ◯ 21 -30
- ◯ 31 - 40
- ◯ 41 - 50
- ◯ 51 - 60
- ◯ 60 +

4. What is your highest Environmental Health qualification obtained? *

- ◯ National Diploma
- ◯ B. Tech
- ◯ Bachelor degree/ BSC degree
- ◯ Master degree
- ◯ Doctoral degree

5. Are you working as an Environmental Health Practitioner in South Africa? *

- ◯ Yes
- ◯ No

6. If you are registered with HPCSA, under which category are you registered? *

- ◯ Student EHP
- ◯ Community Service EHP
- ◯ Independent Practitioner
- ◯ Retired Practitioner

7. Which province are you working in? *

- ◯ Eastern Cape
- ◯ Western Cape
- ◯ Northern Cape
- ◯ KwaZulu Natal
- ◯ Free State
- ◯ North West
- ◯ Gauteng
- ◯ Mpumalanga
- ◯ Limpopo

8. Which municipal category are you employed under? *

- ◯ Metropolitan Municipality
- ◯ District Municipality
- ◯ Local Municipality
- ◯ Non-municipal organization

9. Which post level are you currently at as Environmental Health Practitioner *

- ◯ Environmental Health Practitioner
- ◯ Senior Environmental Health Practitioner
- ◯ Chief Environmental Health Practitioner
- ◯ Manager Environmental health Practitioner

10. Are you rendering municipal health services, as part of your scope? *

- ◯ Yes
- ◯ No
- ◯ Partially

11. If partially on question 10, please specify those partial municipal health services rendered.

12. Do you implement surveillance and prevention of communicable diseases function in your area? *

- ◯ Yes
- ◯ No

13. if yes, which of the mentioned activities do you implement in your area? *

- [ ] Promoting health and hygiene aiming at preventing communicable diseases
- [ ] Collecting, analyzing, and disseminating epidemiological data
- [ ] Educating community members on prevention and control measure of diseases
- [ ] Conducting epidemiological surveillance of diseases
- [ ] Other activities
- [ ] Not Applicable

14. Please specify other activities if you ticked yes on other activities:

15. How do you implement the activities specified on question 13? *

- ○ Continuously as a routine function
- ○ When need arise
- ○ No plan in place
- ○ No implementation
- ○ Not applicable

16. Is there a standard operating procedure or guiding principle for the implementation of activities mentioned in question 13? *

- ○ Yes
- ○ No
- ○ Not applicable

17. If yes to question 16, please specify the SOP or guiding principle thereof (if not applicable specify): *

18. Specify what type of health promotion and hygiene activities do you conduct: *

19. How do you conduct activities on question 18? *

20. Do you collect epidemiological data? *

- ◯ Yes
- ◯ No
- ◯ Not applicable

21. If yes to question 20, specify the types of data you collect for surveillance of diseases *

22. Indicate the method or process used for collecting diseases surveillance data: *

23. Which sources are you visiting to collect disease surveillance data in your area? *

- ☐ Health Facilities
- ☐ Social Media
- ☐ Other
- ☐ Not applicable

24. If your answer includes other on the previous question, please specify all sources you visits: *

25. How often do you visit the mentioned places *

- ◯ Daily
- ◯ Weekly
- ◯ Monthly
- ◯ Bi-Monthly
- ◯ Quarterly
- ◯ Yearly
- ◯ During disease outbreaks
- ◯ Not ongoing
- ◯ Not applicable

26. What guides the frequency of your data collection? *

- ◯ Bylaws
- ◯ Norms and standards for EHPs
- ◯ SOPs
- ◯ Surveillance policy
- ◯ None
- ◯ Other
- ◯ Not applicable

27. If other, please specify: *

[ ]

28. Do you analyze and interpret the collected disease surveillance data? *

- ◯ Always
- ◯ Never
- ◯ Very often
- ◯ Seldom
- ◯ Not applicable

29. How do you analyze and interpret collected data, including the methods you use? *

30. Do you disseminate analyzed data? *

- ◯ Yes
- ◯ No
- ◯ Sometimes
- ◯ Not applicable

31. If yes to question 30, how do you disseminate your data? *

32. Is there an established disease surveillance and information system in your Province? *

- ◯ Yes
- ◯ No

33. Are there any developed environmental health measures, protocols, with reference to prevention of epidemics, emergencies and communicable diseases affecting your population? *

- ◯ Yes
- ◯ No

34. If yes to question 33, please specify: *

35. How often do you educate community members on prevention and control measure of diseases? *

- ◯ Daily
- ◯ Weekly
- ◯ Monthly
- ◯ Bi-Monthly
- ◯ Quartery
- ◯ Yearly
- ◯ During disease outbreaks
- ◯ Not ongoing
- ◯ None
- ◯ Other

36. If other, then specify your frequency: *

37. Where do you conduct education of the community? *

- ☐ Schools
- ☐ Health facilities
- ☐ Community social gatherings
- ☐ Business premises
- ☐ Pensions pay points
- ☐ Other
- ☐ None

38. If other, specify the areas where you conduct education *

This content is neither created nor endorsed by Microsoft. The data you submit will be sent to the form owner.

Microsoft Forms
